# Andrade-Oliveira Salvianolic Acid B Modulates Caspase-1–Mediated Pyroptosis in Renal Ischemia-Reperfusion Injury *via* Nrf2 Pathway

**DOI:** 10.3389/fphar.2020.541426

**Published:** 2020-09-03

**Authors:** Yu Pang, Pei-chun Zhang, Rui-rui Lu, Hong-lian Li, Ji-cheng Li, Hong-xin Fu, Yi-Wen Cao, Guo-xing Fang, Bi-hao Liu, Jun-biao Wu, Jiu-yao Zhou, Yuan Zhou

**Affiliations:** ^1^School of Pharmaceutical Sciences, Guangzhou University of Chinese Medicine, Guangzhou, China; ^2^Department of Urology, The Sixth Affiliated Hospital of Sun Yat-Sen University, Guangzhou, China; ^3^Guangdong Institute of Gastroenterology, The Sixth Affiliated Hospital of Sun Yat-Sen University, Guangzhou, China; ^4^Department of Clinical Pharmacy, The Second Affiliated Hospital of Guangzhou University of Chinese Medicine, Guangzhou, China

**Keywords:** acute renal injury, Salvianolic acid B, pyroptosis, Nrf2/NLRP3 signaling pathway, NLRP3 inflammasome

## Abstract

Acute kidney injury (AKI) is a serious disease characterized by a rapid decline in kidney function. Oxidative stress is the primary pathogenesis of AKI. Salvianolic acid B (SalB), a water-soluble compound extracted from *Salvia miltiorrhiza*, possesses a potent antioxidant activity. Here, we investigated the protective effect of SalB against renal ischemia-reperfusion injury (I/R) in mice. Briefly, by analyzing renal function, oxidative stress markers and inflammatory biomarkers, we found that SalB could improve kidney damage, reduce oxidative stress and inflammatory factor levels. Interestingly, the expression of the NLR family pyrin domain-containing 3 (NLRP3), caspase-1, pyroptosis related proteins gasdermin D (GSDMD) and interleukin (IL)-1β, which were significantly upregulated in the kidney tissues of I/R group, was effectively reversed by SalB. Meanwhile, renal tubular epithelial cells hypoxia and reoxygenation model was used to explore pyroptosis of caspase-1-dependent. Further mechanism study showed that the SalB pretreatment could promote the increase of nuclear factor erythroid-2 related factor 2 (Nrf2) nuclear accumulation, which significantly suppressed oxidative stress, proinflammatory cytokines, NLRP3 inflammasome activation and pyroptosis. These results indicate that SalB can inhibit caspase-1/GSDMD-mediated pyroptosis by activating Nrf2/NLRP3 signaling pathway, resulting in alleviating I/R injury in mice.

## Introduction

Acute kidney injury (AKI) is a severe disease that has a high prevalence and can even cause death in hospitalized patients (Velez et al., 2020). It is a clinical syndrome characterized by a sudden decline in renal function accompanied by cumulative nitrogenous compounds (urea nitrogen and creatinine) with reduced urine output. If not treated properly, it can lead to the occurrence of acute renal failure (ARF) (Kanagasundaram, 2015). Although the pathogenesis of AKI remains unclear, injury or death of renal tubular epithelial cells and inflammation have been identified as key causative factors of AKI (Andrade-Oliveira et al., 2019; Guzzi et al., 2019). In AKI, renal tubular epithelial cells are damaged or perished due to different etiologies. These cells may secrete a signal that triggers a nephritic response, which ultimately leads to the development of ARF (Rabb et al., 2016).

Several studies have shown that signals associated with pyroptosis have a central role in the occurrence and development of AKI (Krautwald and Linkermann, 2014; Kers et al., 2016). Pyroptosis is a proinflammatory programmed cell death different from apoptosis and necrosis (Liu and Sun, 2019), characterized by the release of proinflammatory cytokines and intracellular content (Fernandes-Alnemri et al., 2007; Shi et al., 2017). Pyroptosis is rapidly achieved through two pathways: the classical caspase-1 dependent pathway (Schneider et al., 2017), and the caspase-11–dependent nonclassical secretory pathway (Kayagaki et al., 2015). Specifically, in the classical caspase-1 dependent pathway, caspase-1, also known as pyrophosphate, is a large cysteine-dependent protease. Active caspase-1 has a specific structure of heterotetramers that mediate proteolytic processes of inflammatory and inflammatory cytokines, including interleukin-1β (IL-1β) and IL-18 (Vanaja et al., 2015). Gasdermin D (GSDMD) is cleaved into two fragments by active caspase-1: GSDMD N-terminal fragment (GSDMD-N) and GSDMD C-terminal fragment (GSDMD-C) (Shi et al., 2015). The cell membrane forms membrane pores through the insertion and permeabilization process of GSDMD-N, thereby inducing higher inflammatory cytokine release (Liu X. et al., 2016; Rogers and Alnemri, 2019). Pyroptosis has been reported to act primarily on phagocytic cells, macrophages, monocytes and dendritic cells (Miles et al., 2013; de Almeida et al., 2015; Martinet et al., 2019), as well as various other cell types in inflammatory diseases such as T cells (Luo et al., 2019). Previous studies have shown an enhanced expression of GSDMD in the serum of patients with rheumatoid arthritis (RA) and emphasized pyroptosis in association with RA (Wu et al., 2020). Moreover, Wang Y. et al. (2019) found that chemical GSDMD-related pyroptosis of tubular cells in diabetic kidney disease is dependent on the TLR4/NF-κB signaling pathway. At the same time, studies have emphasized that the loss of renal tubular epithelial cells leads to an increase in renal tubular damage in kidney disease. These findings collectively implicate an important pathogenesis of pyroptosis in AKI, indicating that the improvement of pyroptosis may serve as a potential therapeutic target for AKI.

Reactive oxygen species (ROS) accumulation and inflammation are the key factors causing AKI. Growing evidence has indicated that the expression of inflammatory bodies and ROS in AKI is significantly increased (Sun T. et al., 2019; Yu et al., 2020). With the development of AKI, a large amount of ROS are produced, which leads to the activation of The NLR family pyrin domain-containing 3 (NLRP3) inflammasome and the cleavage of caspase-1, thereby promoting the maturation of inflammatory cytokines and promoting the release of interleukin (IL)-1β and IL-18 (Kim et al., 2019; Zahid et al., 2019). The activation of cl-caspase-1 will induce caspase-1–mediated pyroptosis and aggravate renal injury (Wang et al., 2020; Zhu et al., 2020). Nuclear factor erythroid-2 related factor 2 (Nrf2), which is a well-known transcription factor with an important role in cytoprotection, is activated under stress conditions when excessive ROS are detected (Ungvari et al., 2019; Tsushima et al., 2020). Previous studies have shown that the Nrf2/*NF*-κ*B* pathway improves acute lung injury (Xu et al., 2019). Interestingly, a recent study has reported that the activation of Nrf2 negatively regulates NLRP3 inflammasome activation in kidney injury (Shahzad et al., 2016; Li et al., 2019). In other pathways, Nrf2 dissociates from the Kelch-like ECH-associated protein 1 (Keap1) under oxidative stress, and modulates the thioredoxin-interacting protein-thioredoxin1 (TXNIP-TRX1) complex formation [TXNIP is considered an upstream partner protein to NLRP3 by Bai et al. (2019)], while exerting the regulation effect of NLRP3 inflammasome (Wang C. Y. et al., 2019). ROS is closely related to the activation of NLRP3 inflammasome. Therefore, Nrf2 activation can inhibit ROS-induced activation of NLRP3 inflammasome.

Salvianolic acid B (SalB) is a water-soluble component of the traditional Chinese medicine *Salvia miltiorhiza Bge*, which has various biological activities such as antioxidant, antiinflammatory, antitumor and renal protection (Zhang et al., 2017; Zhao et al., 2017; Huang et al., 2019). Previous studies have suggested that SalB reduces various organ injuries, and maintains the redox homeostasis, especially the balance of ROS (Tang et al., 2014). It has been reported that SalB provides protection by upregulating the Nrf2 antioxidant signaling pathway in animal model (Liu B. et al., 2016; Liu M. et al., 2018; Zhang et al., 2018; Liao et al., 2020). However, there is still no evidence confirming whether SalB protects against AKI through the Nrf2/NLRP3 mechanism. Though pyroptosis may be an essential mechanism underlying the development of AKI in renal tubular cells, it has been addressed by only a few studies, and the data on the relationship between SalB and AKI pyroptosis is even scarcer. Therefore, the aim of this study was to investigate the potential role of pyroptosis during I/R-induced mouse model of AKI and explore the molecular mechanism related to Nrf2/NLRP3 pathway underlying the effects of SalB.

## Materials and Methods

### Animals

Sixty male Balb/c mice, 8–10 weeks old, weighing 18–22 g, were obtained from the Guangzhou University of Chinese Medicine Research Center (License number: SCXK (Guangdong) 2018-0034; Guangzhou, China) and were housed in an SPF animal laboratory (License number: SYXK (GZ) 2018-0085). All the animals were housed in an environment with a room temperature (RT) of 25 ± 1°C, a relative humidity of 65 ± 1%, and a light/dark cycle of 12/12 h. All animal studies (including the mice euthanasia procedure) were done in compliance with the regulations and guidelines of the animal ethics committee of Guangzhou University of Chinese medicine institutional animal care and conducted according to the AAALAC and the IACUC guidelines.

### Renal Ischemia-Reperfusion Model and SalB Treatment

After 1 week of acclimatization, the mice were divided into five groups: sham operation control group (Sham), model group (I/R), the SalB high-dose group (SalB-H), the SalB medium-dose group (SalB-M), and the salB low-dose group (SalB-L); there was no difference in food consumption between the five groups. Except for the sham group, all other groups were surgically modeled. One week before the modeling, the high, medium and low doses of SalB (purity>98%; 358153; Nanjing DASF Biotechnology Co.Ltd.) were intragastrically administered at a dose of 200 mg/kg, 100 mg/kg, 50 mg/kg, respectively, and the sham operation group was given appropriate physiological saline. The dose of SalB was decided according to the previous study (Huang et al., 2016; He et al., 2020) Mice were anesthetized with 1.5% pentobarbital sodium (0.1 ml/100 g weight) and unilateral renal ischemia was induced by microvascular forceps around the left renal pedicle for 45 min, including contralateral nephrectomy. Sham-operated control mice were subjected to an abdominal incision but did not undergo clamping of the renal pedicles. The abdominal incision was closed with suture. After 24 h of surgery, the mice were euthanized, serum and kidney tissue was harvested for analysis.

### Renal Function Tests

The renal function was analyzed by measuring blood urea nitrogen(BUN) and serum creatinine (Scr) using the assay kits (Jiancheng Biotech, Nanjing, China), according to the manufacturer’s instructions.

### Oxidative Stress Analysis

To analyze intracellular oxidative stress, the malondialdehyde (MDA), superoxide dismutase (SOD) and glutathione (GSH) was detected according to the manufacturer’s instructions (Jiancheng Biotech, Nanjing, China).

### Western Blot

Renal tissue and renal tubular epithelial cells were lysed in RIPA lysis buffer on ice, total proteins were quantified by BCA protein quantitative kit (CW0130S; CWBIO) to obtain the protein concentration. Samples were then transferred to polyvinylidene fluoride membrane by 8%–15% SDS-PAGE. The resulting blots were blocked with 5% nonfat milk dissolved in PBS for 3 h and incubated with anti-GSDMD antibody (96458S; CST and sc-393656; Santa Cruz), anti-caspase-1 antibody (ab1872; Abcam), anti-NLRP3 antibody (15101S; CST), anti-IL-1β antibody (12242; CST), anti-Nrf2 antibody (ab137550; Abcam), anti-Keap1 antibody (8047; CST), anti-HO-1 antibody (ab13248; Abcam), anti-TXNIP antibody (14715; CST), at 4°C overnight. The next day, after the blots were washed three times with PBS, and then incubated in horseradish peroxidase-conjugated secondary antibodies for 1 h. The specific proteins were detected by the chemiluminescence (ECL) system.

### Cell Model

Human renal tubular epithelial cells (HK-2) was purchased from the Cell Bank of the Chinese Academy of Sciences. The cells were cultured in DMEM/F12 (SH30023.01B,HyClone) medium containing 10% fetal bovine serum (10099141; Gibco) and 1% penicillin-streptomycin(15140122; Gibco) in a humidified atmosphere containing 5%CO_2_/95% air at 37°C. After two–four passages, cells were cultured for 24 h and then were randomly divided into three groups: Control group: cells were cultured under normal conditions (5% CO2, 21% O2, 74% N2); Model group: the cells were incubated with different concentrations of SalB(purity>98%; 358153; Nanjing DASF Biotechnology Co.Ltd.) for 24 h; Positive control group: cells were incubated in certain concentration of NLRP3 inhibitor MCC950(S7809;Selleck), caspase-1 specific inhibitor VX-765(S2228;Selleck) for 2 h, and were then placed in a hypoxia incubator chamber (27310, stemcell) hypoxia (5% CO2, 95% N2) for 6 h and then reoxygenated for an additional 1 h (5% CO2, 21% O2, 74% N2). The model establishment was decided according to the previous studies (Liu et al., 2019; Yuan et al., 2019).

### Immunofluorescence

After incubation in hypoxia condition for 6 h, cells were fixed with 4% buffered paraformaldehyde for 10 min, permeabilized with 0.5% TritonX-100 for 20 min at RT, rinsed with PBS three times, and blocked with goat serum for 30 min. Cell slides were then incubated with primary antibody against caspase-1, ASC (sc-271054; Santa Cruz) and Nrf2 at 4°C overnight. Consequently, the slides were washed with PBS and incubated with AlexaFluor 555 (4413S; CST) and 488-labeled (4408S; CST)secondary antibodies for 1 h at RT in the dark. All samples were then incubated with DAPI for 5 min and blocked with an antifluorescence quencher. Images were captured using a laser scanning confocal microscope (LSM800;ZEISS).

### Immunohistochemical Staining

The slides were blocked with 3% hydrogen peroxide at RT for 10 min, and the primary antibody anti-caspase-1 was placed in a wet box at 4°C for 16 h. The cells were washed three times with PBS, followed by incubated with horseradish peroxidase at RT for 10 min. The caspase-1 positive staining was visualized with diaminobenzidine (DAB) and the cell nucleus was stained with hematoxylin. After dehydration, slides were mounted using the Rhamsan gum and observed under a microscope.

### Hematoxylin and Eosin Staining

The kidney tissues were collected and embedded in 4% paraformaldehyde. After 48 h, they were embedded in paraffin, cut into 4 μm sections, stained with hematoxylin and eosin (H&E) reagent, and observed under a microscope.

### RNA Extraction and Quantitative Real-Time Polymerase Chain Reaction

Total RNA was extracted from renal tissue using RNAiso Plus (9108,Takara), and its concentration was measured by a nucleic acid-protein analyzer after purification. The RNA was reverse transcribed into cDNA using Takara RT-PCR kit (RR047A;Takara), and then cDNA was mixed with Takara SYBRq PCR kit (RR820A;Takara) to quantify mRNA levels of IL-1β, TNF-α. The RT-PCR reaction was performed for 40 cycles in the ABI 7500 system, with GAPDH as an internal reference and mRNA levels calculated by the 2^-ΔΔCT^ method.

### Scanning Electron Microscope

HK-2 cells were seeded into six-well plates. After reaching 70% confluence, cells (except the Sham group) were pre-treated with SalB, followed by the H/R treatment. Cells were then fixed with ice-cold 2.5% glutaraldehyde at 4°C for 24 h. After elution in a series of concentrations of ethanol and desiccation by isoamyl acetate, cells were sputtered coating with lon-sputtering instrument. Specimens were detected using a scanning electron microscope (SU8010; Hitachi, Tokyo, Japan).

### Detection of the Frequency of Annexin V+/PI+ by Flow Cytometry

Cell death was analyzed by flow cytometry (BD FACSCanto II, USA) using the Annexin V-FITC/PI Apoptosis Detection Kit (Beyotime, JiangSu, China). In brief, the cells from six groups were harvested and washed twice with PBS. Cells were then stained with 10μl Annexin V-FITC and 10 μl propidium iodide in the dark. Consequently, cells were detected with flow cytometry and analyzed with FlowJo 7.6 software (FlowJo, Ash-land, OR, USA).

### Measurement of Cellular ROS Levels

Cell superoxide level was determined by DCFH-DA (BestBio, Shanghai, China). HK-2 cells were exposed with different stimulations and incubated with DCFH-DA for 60 min in a light-protected humidified chamber and washed. The fluorescence intensity of DCFH-DA was measured by the laser scanning confocal microscope.

### Small Interfering RNA Transfection

Small interfering RNA transfection Nrf2 siRNA was transformed by ribo FECTTM CP reagent (Ribobio, Shanghai, China) according to the manufacturer’s protocol. The sequence of Nrf2 siRNA was 5′- GAGAAAGAATTGCCTGTAA -3′.

### Statistical Analyses

Statistical Analysis Continuous variables are presented as the mean ± S.E.M. Analysis of variance (ANOVA) and *post hoc* Bonferroni analysis was conducted for multiple comparisons by SPSS software-version 20.0. A *P*-values<0.05 indicated a statistically significant difference.

## Results

### The Renoprotective Effects of SalB on I/R Mice

**Figure 1:**
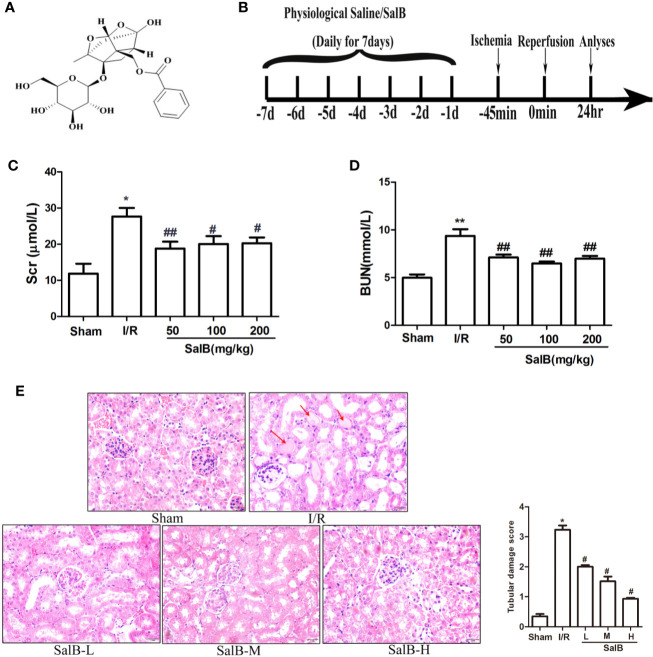
SalB treatment ameliorates renal function and renal tubule pathological injury induced by ischemia-reperfusion (I/R). **(A)** The chemical structure of Salvianolic acid B (SalB). **(B)** Experimental design. **(C, D)** Serum creatinine and blood urea nitrogen levels. The data show means ± SEM (n = 6).*P < 0.05, **P < 0.01 vs. sham group; ^#^P < 0.05, ^##^P < 0.01 vs. I/R group. **(E)** Kidney tissue sections were subjected to histological examination by hematoxylin and eosin staining (H&E) to evaluate renal tubule injury; protein casts are shown with the red arrow. Tubular damage was scored in a double-blind manner method based on the percentage of injury included tubular dilation and intertubular hemorrhage: 0, no damage; 1, < 25%; 2, 25 ~ 50%; 3, 50 ~ 75%; 4, > 75%) Magnification: 400×.

SalB is one of the effective medicinal ingredients of *Salvia miltiorrhiza Bge*, which is formed by the condensation of three molecules of Danshensu and one molecule of caffeic acid (**Figure 1A**). To investigate the possible effects of SalB on AKI, a mouse model of AKI was induced by I/R (**Figure 1B**). The levels of serum Scr and BUN were assessed and HE staining was performed. Compared with the sham group, the Scr and BUN levels were significantly increased in the I/R group, which indicated a decrease in renal function in I/R mice (**Figures 1C, D****)**. It was improved after treatment with SalB, and the difference between the doses is not obvious. HE staining showed that I/R surgery resulted in severe AKI, which was characterized by vacuolization of the renal tubules, transparent tube type in the lumen and red-stained cytoplasmic granules in I/R mice (**Figure 1E**). Significantly, renal tubular damage was reduced in the mice treated with SalB. The damage index analysis shows that there is a dose-dependent relationship after SalB treatment. Besides, the improvement effect is significant in the high-dose group. These results indicated that SalB improves AKI in I/R mice.

### SalB Inhibits Pyroptosis In Vivo and In Vitro

**Figure 2:**
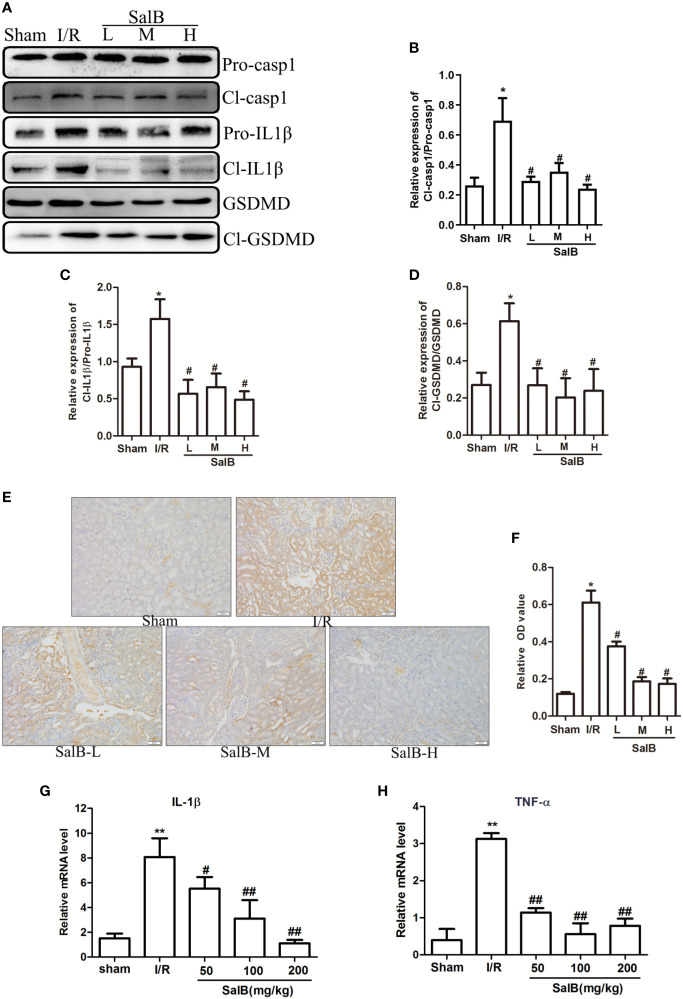
SalB treatment reversed the effect of pyroptosis related genes in ischemia-reperfusion (I/R) mice. **(A–D)** Western blot analysis of the active cleaved form of caspase-1 (Cl-casp1), proform of caspase-1 (Pro-casp1), active cleaved form of interleukin-1β (IL-1β) (Cl-IL-1β), proform of IL-1β (Pro-IL-1β), gasdermin D (GSDMD), GSDMD-N terminal segment (Cl-GSDMD). The data are presented as the mean ± SEM (n = 3), *P < 0.05, **P < 0.01 vs. sham group; ^#^P < 0.05, ^##^P < 0.01 vs. I/R group. **(E, F)** Immunohistochemical staining and quantitative analysis for caspase-1 was shown (n = 6, magnification: 400×). **(G, H)** mRNA levels in renal tissues of IL-1β and TNF-α determined by real-time polymerase chain reaction (PCR). The data are means ± SEM (n = 6).*P < 0.05, **P < 0.01 vs. sham group; ^#^P < 0.05, ^##^P < 0.01 vs. I/R group.

Next, we assessed whether pyroptosis contributed to the progression of AKI and had an important role in this process. The protein expression levels of pyroptosis related proteins GSDMD, caspase-1 and IL-1β in the kidney tissues were measured. Protein expression of active cleaved form of caspase-1 (Cl- casp1), proform of caspase-1 (Pro-casp1), active cleaved form of IL-1β (Cl- IL1β), proform of IL-1β (Pro-IL-1β), GSDMD, GSDMD-N terminal segment (Cl-GSDMD) were strongly upregulated in the I/R group, which was effectively reversed by SalB (**Figures 2A–D**). Immunohistochemical staining of caspase-1 produced another evidence of pyroptosis in the renal tubule, and histological analysis of the kidney showed a significant increase in caspase-1 activation (**Figures 2E, F**). Besides, the I/R group significantly increased the expression of pyroptosis, which was reflected by a sustained increase in IL-1β and TNF-α mRNA levels (**Figures 2G, H**). Therefore, SalB significantly inhibited the expression of pyroptosis in I/R mice.

**Figure 3:**
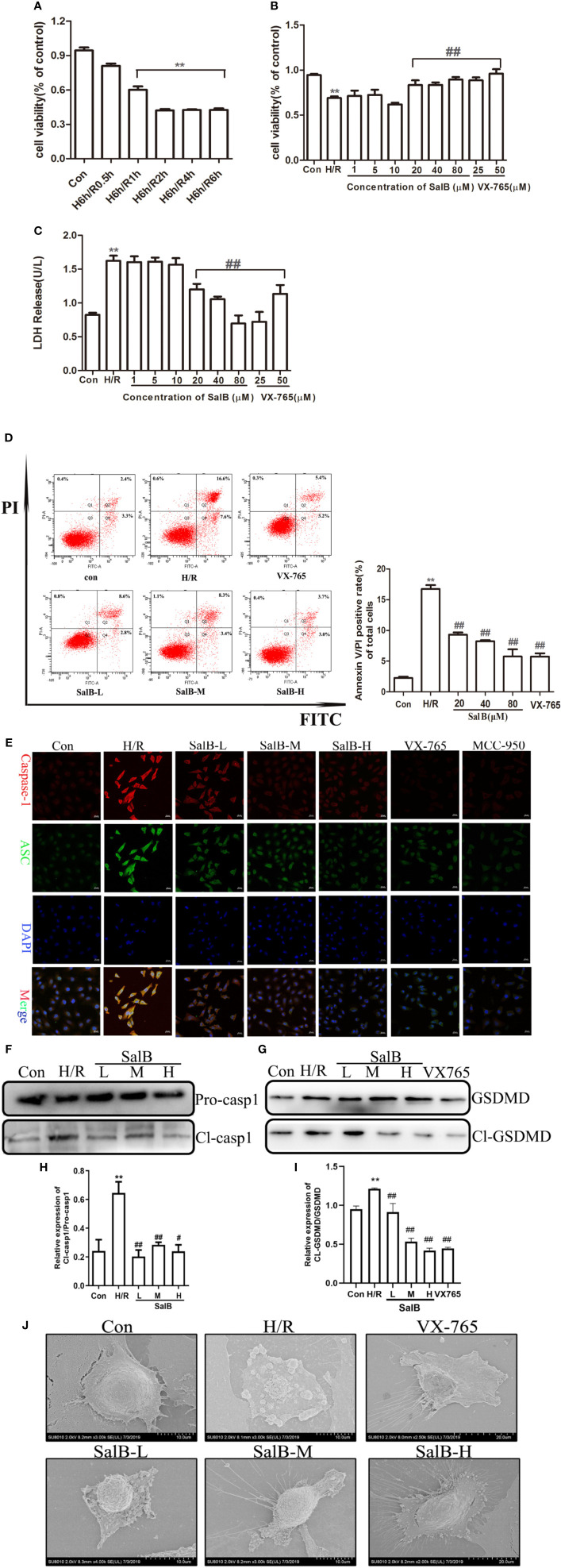
Salvianolic acid B (SalB) down-regulation the expression of pyroptosis in H/R-induced HK-2 cells. **(A)** HK-2 cells treated with 6 h of hypoxia and 0.5, 1, 2, 4, and 6 h of reoxygenation was measured using an MTT kit. **(B)** Different concentrations of SalB (1, 5, 10, 20, 40, and 80 μM) was measured using an MTT kit. **(C)** The level of LDH release. **(D)** The frequency of caspase-1+PI pyroptotic of HK-2 cells was analyzed by flow cytometry. **(E)** Immunofluorescence staining results of the expression of caspase-1 and ASC (magnification ×400). Blue: nuclear staining (DAPI); green: ASC; red: Capsase-1. Scale bar: 20 μm. **(F–I)** Protein levels the cleaved form of caspase-1 (Cl- casp1), proform of caspase-1 (Pro-casp1), GSDMD, GSDMD-N terminal segment (Cl-GSDMD). Data from three separate experiments are represented as images or are expressed as the mean ± SEM of each group (n = 3 per group for *in vitro* assay) **P < 0.01 versus control group; ^#^P < 0.05 ^##^P < 0.01 versus H/R group. **(J)** After SalB treatment, representative scanning electron micrographs of the HK-2 cells obtained sections of control and H/R group. Scale bars: 2 μm (upper) and 0.5 μm (lower).

Renal tubular cells were identified as the primary site of AKI, and data indicated that lesion death occurred in renal injury induced by I/R. To further determine the mechanism of SalB in AKI, HK-2 was used for H6h/R1h treatment to induce pyroptosis. Cell viability was significantly inhibited after H/R treatment compared to control (**Figure 3A**). In addition, the cell viability was significantly improved at the concentrations of 20μM, 40μM and 80μM, (**Figure 3B**), based on which these three concentrations were chosen for subsequent experiments. Moreover, H/R treatment increased LDH content in HK-2 cell supernatants compared to control (**Figure 3C**). Also, we found that H/R treatment significantly increased the PI double positive rate of HK-2 cells (**Figure 3D**). Besides, both of ASC (an apoptosis-associated speck-like protein containing CARD) and caspase-1 are the components of the inflammasome. The immunofluorescence results showed that the expression of caspase-1 and ASC protein in HK-2 cells after H/R treatment was significantly enhanced (**Figure 3E**). Consistent with these findings, H/R treatment significantly increased the protein levels of HK-2 cells cl-caspase-1, caspase-1, cl-GSDMD, GSDMD (**Figures 3F–I**). A scanning electron microscope confirmed the presence of pyroptosis. H/R-induced pyroptosis in HK-2 cells was identified, through the formation of pores on the cell membrane, which leads to loss of its integrity. In addition, the contents were released, causing an inflammatory reaction compared with the control group. At this time, the nucleus was located in the center of the cell (**Figure 3J**). These results showed that pyroptosis was similarly induced in renal tubules HK-2 cells by simulating I/R with H/R model.

### Nrf2/NLRP3 Signaling Pathway Is Involved in the Inhibition of Pyroptosis in AKI by SalB

**Figure 4:**
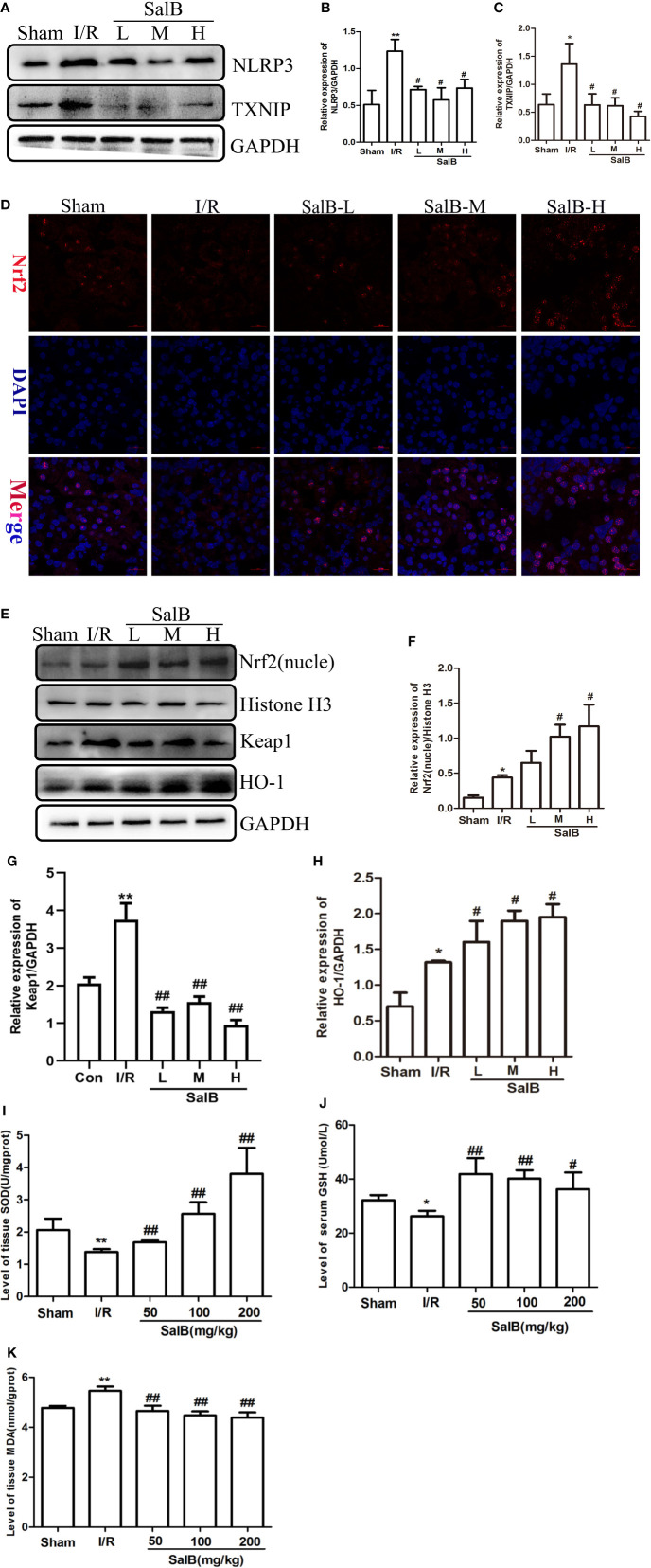
Salvianolic acid B (SalB) promotes Nrf2 nuclear activation and inhibits NLR family pyrin domain-containing 3 (NLRP3)/thioredoxin-interacting protein-thioredoxin1 (TXNIP) expression in ischemia-reperfusion (I/R) mice. **(A–C)** The expression of NLRP3 and TXNIP. **(D)** Immunofluorescence images (magnification ×200) showing the nuclear expression and localization of Nrf2 in the Sham, I/R, SalB-L, SalB-M, SalB-H groups. Blue: nuclear staining (DAPI); red: Nrf2; staining. Scale bar: 20 μm. **(E)** Representative western blots and **(F–H)** quantification of relative protein expression for nuclear Nrf2, keap1 and HO-1. **(I–K)** Superoxide dismutase (SOD), glutathione (GSH), and malondialdehyde (MDA) detected by a microplate reader. Data are represented as images or expressed as the mean ± SEM of each group from three separate experiments. *p < 0.05, **p < 0.01 vs. sham group; ^#^p < 0.05, ^##^p < 0.01 vs. I/R group.

**Figure 5:**
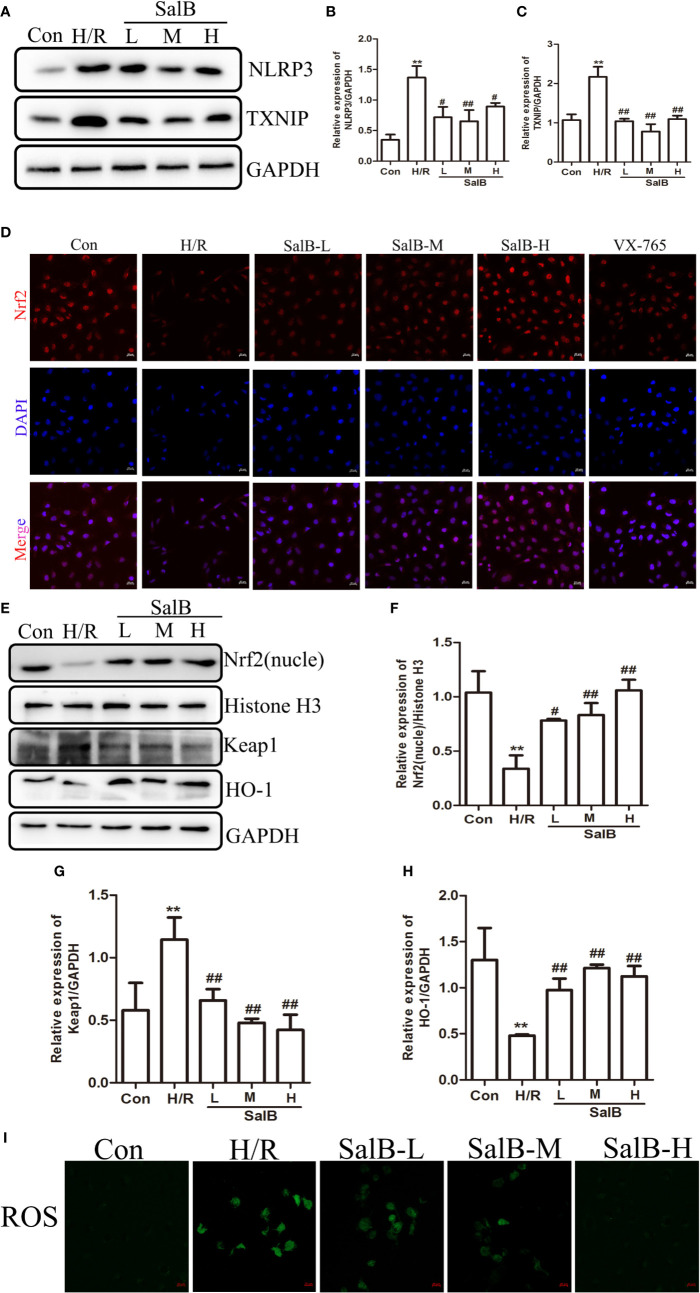
Nuclear factor erythroid-2 related factor 2 (Nrf2) nuclear expression is upregulated and NLR family pyrin domain-containing 3 (NLRP3)/thioredoxin-interacting protein-thioredoxin1 (TXNIP) is down-regulated after SalB treatment in H/R **(A–C)** NLRP3 and TXNIP expression were examined by western blot. **(D)** Immunofluorescence results (magnification ×400) showing the expression of Nrf2 under normal conditions (control), SalB treatment and H/R-treated HK-2 cells. Blue, nuclear staining (DAPI); red, Nrf2 staining. Scale bar: 20 μm. **(E)** Representative western blots and **(F–H)** quantification of relative protein expression for nuclear Nrf2, keap1 and HO-1. **(I)** Representative images of fluorescence of ROS probed by DCFH-DA. Data are represented as images or expressed as the mean ± SEM of each group from three separate experiments. **p < 0.01 vs. control group; ^#^p < 0.05, ^##^p < 0.01 vs. H/R group.

We next used immunoblotting to determine the expression levels of NLRP3 in the kidneys. We found that the NLRP3 protein levels were significantly upregulated in the kidneys of I/R mice, which were reversed by SalB treatment. Interestingly, we found that TXNIP protein was also upregulated considerably in I/R (**Figures 4A–C**). Liu et al. have recently reported that the inhibition of TXNIP-TRX1 complex dissociation by Nrf2 activation prevents TXNIP from activating NLRP3 protein. We tried to explore the possible action proteins of SalB that inhibit the expression of TXNIP and NLRP3. Results of immunofluorescence staining and Western blotting showed that SalB could activate Nrf2 nuclear expression and activate HO-1 protein by inhibiting Keap1 protein expression, which indicated that SalB could significantly improve the antioxidant capacity of I/R mice (**Figures 4D–H**). Consistent with these results, the levels of oxidative stress indicators SOD, GSH and MDA were examined, and the results showed that SalB could effectively inhibit oxidative stress levels *in vivo* (**Figures 4I–K**). Consistent with *in vivo* results, SalB inhibited TXNIP and NLRP3 protein expression of HK-2 after H/R treatment (**Figures 5A–C**) and increased Nrf2 nuclear import and activated expression of downstream antioxidant components (**Figures 5D–H**). The signal of fluorescence was blunted in cells subjected to H/R but was markedly increased by SalB treatment present throughout the process of H/R (**Figure 5I**). In conclusion, SalB activates Nrf2 nuclear expression and inhibits TXNIP and NLRP3 protein expression.

### The Effect of Nrf2 Knockdown on the Antioxidative Stress and Antipyroptosis Effects of SalB in H/R

**Figure 6:**
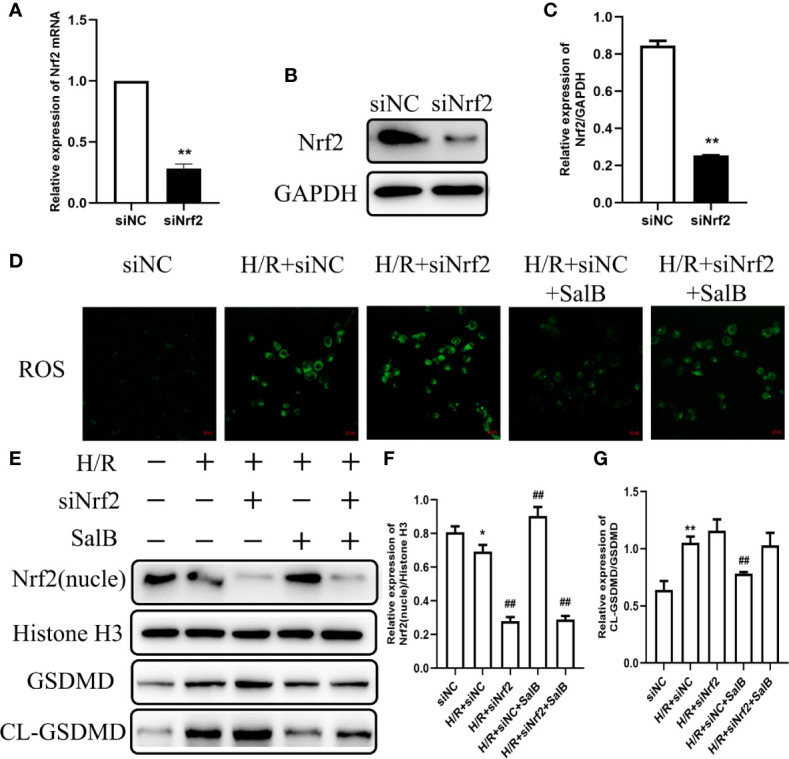
Effects of nuclear factor erythroid-2 related factor 2 (Nrf2) knockdown on H/R induced renal tubular cell damage. HK-2 cells were transfected with siNrf2 for 48 h before treating with Salvianolic acid B (SalB) (80 μM) stimulation for 24 h. **(A–C)** The transfection efficiency of siNrf2 was determined by Q-PCR and western blot. **(D)** Represented images showing the superoxide anions detected by DCFH-DA staining. **(E–G)** The expression of Nrf2, gasdermin D (GSDMD), cl-GSDMD were detected. The data are means ± SEM (n = 3). *P < 0.05, **P < 0.01 versus control group; ^##^P < 0.01 versus H/R group.

In order to explore the mechanism by which SalB regulates cell oxidative stress and pyroptosis, siNrf2 was used. Specific knockdown of Nrf2 in HK-2 cells was confirmed at the mRNA and protein expressions (**Figures 6A–C**). Knockdown of Nrf2 significantly increased the oxidative stress induced by H/R, and the addition of SalB still cannot reduce the fluorescence intensity of DCFH-DA, indicating that the antioxidant effect of SalB is impaired (**Figure 6D**). After siNrf2 transfection, the protein levels of cl-GSDMD/GSDMD in H/R-induced HK-2 cells increased, indicating an increase in pyroptosis. As expected, compared with the treatment group without siNrf2, SalB treatment scarcely changed the expression of these pyroptosis markers in cells pretreated with siNrf2 (**Figures 6E–G**). Therefore, these data proved that SalB exerts an antipyroptosis effect by regulating the Nrf2 pathway in HK-2 cells.

## Discussion

In this study, we investigated the effect of SalB on kidney injury through the I/R animal model and H/R cell culture system. SalB exerts its protective effects by reducing the levels of BUN and SCr. In addition, SalB mediated the inhibition of pyroptosis, which is associated with the activation of NLRP3. Further study demonstrated that SalB inhibited the release of ROS by activation of Nrf2 nuclear translocation, resulting the reduction of inflammation and pyroptosis during kidney injury. our results provided a rational for the use of SalB as a potential supplemental treatment to attenuate kidney injury.

Previous studies have shown that I/R-induced tubular cell death is a major cause of the development and progression of ARF. Necrosis and apoptosis is the main pathway leading to tubular cell death after I/R (Linkermann et al., 2014), but caspase inhibitors that inhibit apoptosis as a target did not completely prevent the AKI process, indicating that there must be other forms of death involved. However, I/R induces a large number of inflammatory reactions and renal tubular cell death, for which the molecular and signaling mechanisms of inflammation remain largely unknown (Tajima et al., 2019). Therefore, it is of significant importance to search for the mechanisms of I/R-induced inflammatory response. Pyroptosis is a type of inflammatory cell death, which depends on inflammatory caspase-mediated cleavage of GSDMD (Jorgensen et al., 2016b). The rapid inflammatory response induced by the occurrence of pyroptosis directly promotes the development of the disease (Liu and Lieberman, 2017). Previous study has revealed that caspase-1 is a key enzyme that could mediate the process of pyroptosis, which means that caspase-1 overexpression may be a hallmark of pyroptosis (Fink and Cookson, 2006; Miao et al., 2011). Activation of caspase-1 not only produces IL-1β a mature inflammatory cytokine (Karmakar et al., 2015; Jorgensen et al., 2016a; Kim et al., 2019), but also causes cell membrane perforation, resulting in the production and release of a large number of inflammatory factors that aggravate pyroptosis. It is reported that GSDMD protein, which critically determines pyroptosis has been identified (Aglietti and Dueber, 2017). Our study showed that the expression of the cleaved-caspase1 and GSDMD-N-terminal fragment protein increased significantly, indicating the occurrence of pyroptosis. Meanwhile, pyroptosis in AKI is accompanied by the inflammatory response, manifesting as the release of inflammatory cytokines (IL-1β and TNF-α).

In our results, the cell viability of HK-2 decreased and LDH content increased, indicating that LDH was released and abundantly present in the extracellular matrix. Besides, SalB significantly restored renal function in I/R mice and inhibited pyroptosis in both I/R and HK-2 cell models. Previous studies used the annexin V and propidium iodide double-positive stage and an LDH-release assay to confirm pyroptosis (Liu W. et al., 2018; Jia et al., 2019); however, the specific cell morphology evidence of pyroptosis was lacking. Over recent years, different studies have recorded the morphology of the cells of pyroptosis by electron microscopy, despite pyroptosis occurring in different cells (HeLa cells (Wang et al., 2017) and raw-asc cells), which all have the similar morphology of pyroptosis. The dying cells showed evident swelling with characteristic bubbling from the plasma membrane, where corpses of pyroptotic cells were described as resembling fried egg (Chen et al., 2016). A large number of pores were mediated by GSDMD-N in pyroptosis, and because of the nonselectivity of GSDMD-formed pores, the intracellular osmotic pressure did not undergo a substantial increase, thus preventing the early pyroptotic cell from bursting. Our data are consistent with those published in previous studies arguing that pyroptosis was triggered in H/R-induced HK-2 cell damage, which resembled the fried egg as shown by electron microscopy in **Figure 3K**, and which occurred at late stage of pyroptosis. Pyroptotic morphology was also improved after treatment with SalB. Further investigation on pyroptosis in AKI will provide a new mechanism for SalB to resist AKI.

NLRP3 inflammasome has been considered to be the link between pyroptosis and inflammation in AKI (Qiu Z. et al., 2019; Sun W. et al., 2019). It has been reported that TXNIP triggers the activation of NLRP3 by binding to NLRP3 after dissociation of the TXNIP-TRX1 complex (Jin et al., 2019). In the inflammatory response of AKI, TXNIP is also considered as a critical link in inflammation (Wen et al., 2018). Our study has demonstrated that SalB can alleviate the proinflammatory effects of NLRP3 and TXNIP. In addition, activation of Nrf2 induces the expression of HO-1 and downstream antioxidant protein, suggesting that Nrf2 is essential for the regulation of HO-1. Previous studies have indicated that the Nrf2 signaling pathway plays a key role in inflammatory responses and oxidative stress during I/R. Nrf2 knockout increases the level of oxidative stress in AKI and exacerbates the ischemic injury, which, when activated, inhibits ROS levels and reduces AKI kidney damage (Rubio-Navarro et al., 2019). Consistently, the increased expression of MDA, SOD and GSH in I/R mice reflects the increase in oxidative stress levels, and SalB treatment has a significant improvement effect, especially in the high-dose group. Normally Nrf2 is captured by Keap1 in the cytoplasm. After being attacked by signals from reactive oxygen species or nucleophiles, Nrf2 dissociates from Keap1, and then the stable Nrf2 translocates into the nucleus. Consistent with our cell experiment, when H6R1, the expression of Nrf2 and Keap1 were inversely proportional. SalB treatment can significantly increase the nuclear expression of Nrf2 and the expression of related antioxidant markers in the H/R model, which indicated that SalB might play a role in improving pyroptosis especially in the high-dose group. According to reports, the expression of Nrf2 will increase after I/R (Li et al., 2018; Qiu Y. et al., 2019). In our study, the expression of Nrf2 increased after modeling. This may be the spontaneous protective activation of the body under stress conditions, and the specific mechanism needs further study. It is worth noting that SalB pretreatment more significantly increased the nuclear expression level of Nrf2, which indicates the antioxidant function of SalB. At the same time, we found that siNrf2 could eliminate the protective effect of SalB. This result further confirms that the participation of Nrf2 plays a key role in the antipyroptosis effect of SalB in AKI.

Collectively, our results revealed that the primary mechanism through which SalB improves AKI is by inhibiting the activation of NLRP3 by direct activation of nuclear expression of Nrf2, thereby inhibiting pyroptosis. These findings illustrated a previously unknown pathway for the SalB in the treatment of AKI, which involves the modulation of Nrf2/NLRP3 signaling and pyroptosis. Yet, it is undeniable that in addition to the Nrf2/NLRP3 signaling pathway, the role of SalB in AKI can be mediated through other mechanisms, which need to be addressed by further research. This study confirmed the existence of pyroptosis in I/R and greatly elucidates the role of SalB can significantly reduce pyroptosis. At the same time, it also reveals the new mechanism of SalB in the treatment of AKI, thus providing a basis for further clinical research.

## Conclusions

**Figure 7:**
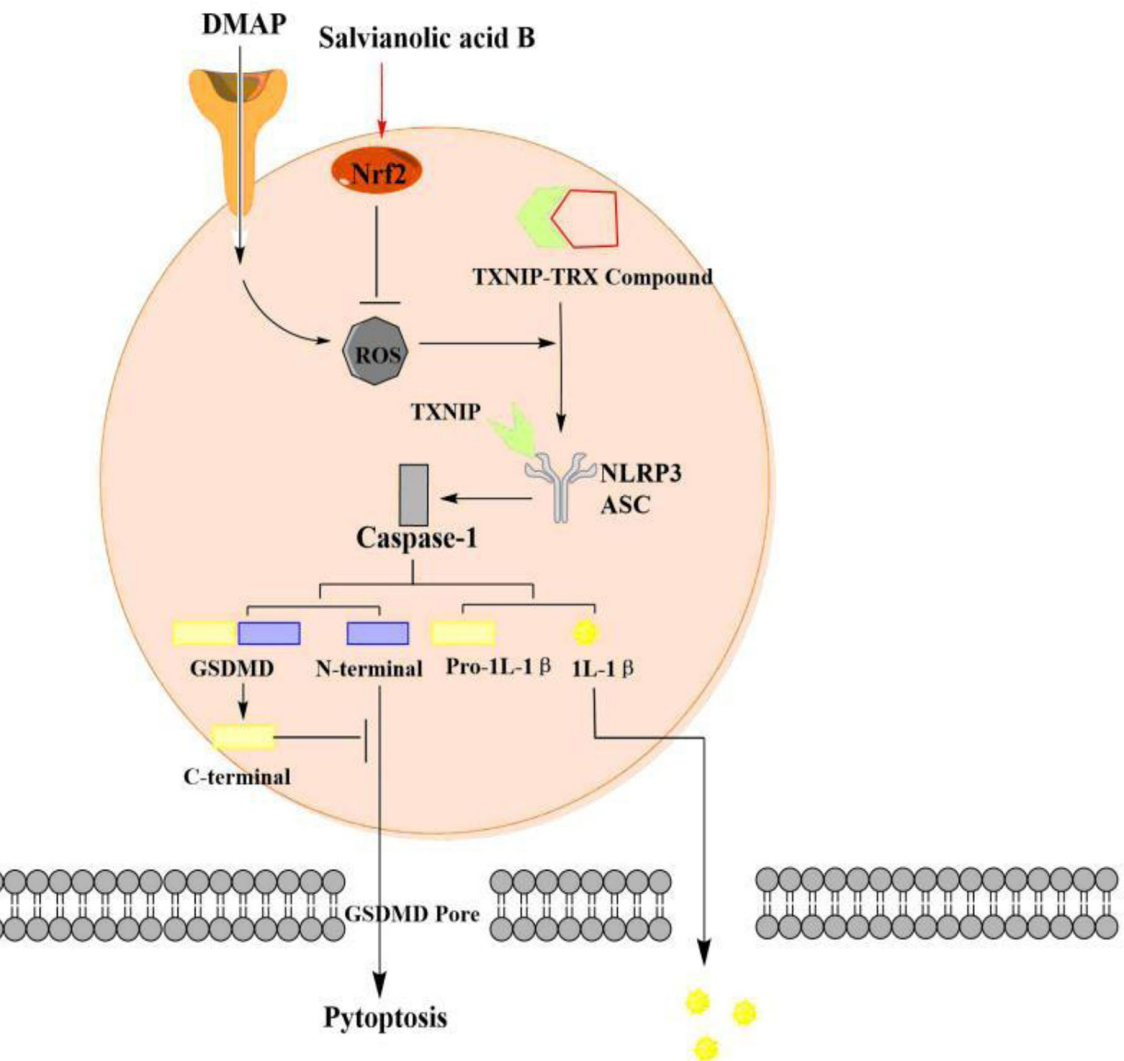
The pyroptosis of acute kidney injury (AKI) signaling and protective effect of SalB through the nuclear factor erythroid-2 related factor 2 (Nrf2)/NLR family pyrin domain-containing 3 (NLRP3) pathway. I/R and H/R trigger pyroptotic cell death signaling, including upregulation of thioredoxin-interacting protein-thioredoxin1 (TXNIP) and suppression of Nrf2 expression, leads to NLRP3 oligomerization, ASC recruitment and subsequent caspase-1 activation. Fortunately, SalB treatment ameliorates AKI by regulating the Nrf2/NLRP3 pathway. The arrows represent promotion, while the inverted T represent inhibition. The effect of Salvianolic acid B (SalB) is shown in red.

In this study, we found that SalB improved AKI by alleviating pyroptosis *in vivo* and *in vitro*. Our results suggested that SalB inhibit the dissociation of TXNIP by activating Nrf2 and preventing the activation of NLRP3, thereby improving pyroptosis in AKI (**Figure 7**). However, this study focused on pyroptosis and confirmed its mechanism. The proportion or time window of pyroptosis and other forms of death during AKI can be discussed and subdivided in future research.

## Data Availability Statement

All datasets generated for this study are included in the article/supplementary material.

## Ethics Statement

The animal study was reviewed and approved by the animal ethics committee of Guangzhou University of Chinese medicine institutional animal care and conducted.

## Author Contributions

P-CZ and YP conceived the study, designed and performed the majority of experiments, assisted by J-YZ and YZ. The data were analyzed by R-RL and B-HL. J-BW, Y-WC, and G-XF helped draft the manuscript. J-CL, H-LL, and H-XF performed *in vitro* experiments. All authors contributed to the article and approved the submitted version.

## Funding

This study was supported by the National Natural Science Foundation of China (No. 81803824 and No. 81673874), the Natural Science Foundation of Guangdong Province (2018A030313328 and 2018B0303110004), and the Department of Education of Guangdong Province (No.2016KZDXM030).

## Conflict of Interest

The authors declare that the research was conducted in the absence of any commercial or financial relationships that could be construed as a potential conflict of interest.
